# The Cumulative Detrimental Effect of COVID-19 Pneumonia in a Patient with Myasthenic Crisis: A Case Report and Overview of the Literature

**DOI:** 10.3390/life12101482

**Published:** 2022-09-23

**Authors:** Georgiana-Cristina Buzatu, Florin-Teodor Bobirca, Sebastian Isac, Oana Antonia Mihalache, Mihail Cotorogea-Simion, Alina Tita, Cristian Cobilinschi, Maria Daniela Tanasescu, Anca Bobirca, Gabriela Droc

**Affiliations:** 1 Department of Anesthesiology and Intensive Care I, ‘Fundeni’ Clinical Institute, 022328 Bucharest, Romania; 2 Department of General Surgery, Dr. Ion Cantacuzino Hospital, 073206 Bucharest, Romania; 3 Department of Physiology, Faculty of Medicine, Carol Davila University of Medicine and Pharmacy, 020021 Bucharest, Romania; 4 Department of Neurology, Fundeni Clinical Institute, 022328 Bucharest, Romania; 5 Department of Anesthesiology and Intensive Care, Clinical Emergency Hospital, 014461 Bucharest, Romania; 6 Department of Medical Semiology, Discipline of Internal Medicine I and Nephrology, Faculty of Medicine, Carol Davila University of Medicine and Pharmacy, 020021 Bucharest, Romania; 7 Department of Rheumatology, Dr. Ion Cantacuzino Hospital, 073206 Bucharest, Romania

**Keywords:** COVID-19 pneumonia, myasthenic crisis, plasmapheresis, immunoglobulin therapy, immunosuppression, case report

## Abstract

**Simple Summary:**

A patient with myasthenia gravis, and one very recent acute episode, required ICU admission and immunomodulatory therapy (i.v. immunoglobulins). Shortly after remission of the symptoms, the patient was readmitted to the ICU for COVID-19 pneumonia-induced acute respiratory failure. For the worsening muscle weakness, the patient had to receive five additional sessions of plasma exchange therapy. After receiving remdesivir, plasmapheresis, i.v. dexamethasone, and supportive therapies, the patient was transferred to the neurological ward, which he left 20 days later, after rehabilitation, with no detectable long-term consequences.

**Abstract:**

Background: As the COVID-19 pandemic reached its peak, it became unavoidable that patients with other risk factors for severe pulmonary impairment (such as neuromuscular illnesses) would become afflicted. While the subject of myasthenic crisis secondary to COVID-19 pneumonia represents an interesting topic in the literature, we could not find consistent data that include, as a novel therapeutic approach, both intravenous immunoglobulin and plasma exchange therapy for the treatment of these two concurrent diseases. Case summary: A 69-year-old man with known seropositive generalized myasthenia gravis, hypertension, ischaemic heart disease, NYHA class II-III heart failure, cerebrovascular disease, and recurrent urinary tract infections, was admitted to the ICU for mixed acute respiratory failure, elevated serum lactate and liver function enzymes, and severe thrombocytopenia. A SARS-CoV-2 PCR test was positive, despite a previous COVID-19 pneumonia episode, 10 months prior to the current one. The patient had a recent ICU admission for a myasthenic crisis, which required non-invasive mechanical ventilation and intravenous immunoglobulin therapy. He received supportive therapy, as well as etiological (intravenous remdesivir, plasmapheresis and intravenous dexamethasone). Fifteen days after admission, the patient was transferred to the neurological ward, whence he left 20 days later, with no apparent sequelae. Conclusions: Subsequent intravenous immunoglobulins and plasma exchange therapy appear to be effective and safe in patients with simultaneous acute myasthenic episode and COVID-19 pneumonia.

## 1. Introduction

Myasthenia gravis (MG) is a chronic, autoimmune neuromuscular disease, whose pathological trait is the presence of autoantibodies targeting proteins of the neuromuscular junction. The antibodies binding the target proteins, such as the acetylcholine receptor (AChR), the muscle-specific tyrosine kinase (MuSK), the lipoprotein-related protein 4 (LRP4), and other postsynaptic structures, seem to be the cause of the skeletal muscle weakness (ocular, bulbar, limb, respiratory muscles) [1]. There are two major phenotypes: an ocular and a generalized form, with the generalized one representing up to 80–90% of all myasthenia gravis cases [2,3].

The patients with the generalized form are prone to myasthenic crises (MC), which result in the aggravation of the myasthenic deficit, with progressive worsening of muscle weakness. As a result, respiratory failure could occur, requiring intubation and mechanical ventilation [4]. Infectious episodes (including respiratory tract infections) are the most important aggravating factors for MC [5]. The treatment of acute episodes is even more challenging, considering the specific immunosuppressive therapy of these patients [5].

Considering the COVID-19 pandemic, special attention should be given to immunocompromised patients [6,7].

This infection is characterized by an exaggerated inflammatory response which leads, in symptomatic patients, to acute respiratory distress syndrome [ARDS], sepsis, coagulopathy, and, in some cases, death [8]. This exacerbated inflammatory response could be, however, compromised in immunosuppressed patients in MC, with consequent deleterious effects [5].

Recent evidence suggests that COVID-19-associated coagulopathy is a combination of low-grade disseminated intravascular coagulopathy and pulmonary thrombotic microangiopathy, which can lead to pulmonary dysfunction in severely affected patients [9]. Consequently, additional neuromuscular weakness of the upper trunk secondary to MC predisposes patients with COVID-19 pneumonia to a worsened outcome [10].

This case report aims to raise the clinician’s awareness regarding this unique detrimental association between parenchymal lung involvement due to COVID-related pneumonia and muscular respiratory weakness secondary to MC. Moreover, highlighting new therapeutic approaches could impact, in the future, the general prognosis of these patients.

## 2. Case Report

A 69-year old, 75 kg, 175 cm tall Caucasian male was admitted to the intensive care unit (ICU) for mixed respiratory failure due to the onset of a SARS-CoV-2 infection associated with a MC in remission. The patient was not vaccinated against COVID-19. He had recently been admitted to the ICU to undergo intravenous immunoglobulin therapy (IvIG) and non-invasive mechanical ventilation for the aforementioned MC.

His medical records included a seropositive [AChR antibody titer = 25.1 mmol/L] generalized form of MG, with two MC in the previous year, which responded to intravenous immunoglobulin therapy. Repetitive nerve stimulation test result showed a decrement of >10% and the chest CT scan revealed a normal thymus lodge three years prior, when he presented progressive limb weakness, ptosis, and dysphagia. According to the Myasthenia Gravis Foundation of America (MGFA) score, he was classified as 2B and his Myasthenia Gravis Activities of Daily Living (MG-ADL) score was 7.

Additionally, the patient suffered from COVID 19 pneumonia (10 months prior to the current episode), essential hypertension, ischaemic heart disease, New York Heart Association class II–III heart failure, cerebrovascular disease, cataracts, recurring urinary tract infections (one of which was considered to be the triggering factor for the aforementioned myasthenia flare-up).

At admission, the patient was awake and oriented, Glasgow Coma Scale 15 points, complaining of difficulty breathing, with an increased respiratory drive, peripheral blood oxygen saturation = 76% under 10 L/min supplemental oxygen, hemodynamically stable, blood pressure = 125/65 mmHg, sinus tachycardia, heart rate = 105 bpm, with an aggravated myasthenic bulbar deficit, marked difficulty in mobilizing the head and neck, and difficult deglutition. The arterial blood gas sample revealed severe respiratory acidosis, hyperglycemia, anemia, and lactacidemia: pH = 7.12, pCO_2_ = 82 mmHg, Na = 137 mmol/L, K = 4.5 mmol/L, Ca = 1.24 mmol/L, glycaemia = 208 mg/dL, lactate = 5.2 mmol/L, serum bicarbonate = 26 mmol/L, base excess = −2.6 mmol/L, haemoglobin = 8.6 g/dL. Moreover, the patient exhibited a moderate hepatic cytolytic syndrome (aspartate aminotransferase = 90 U/L and alanine aminotransferase = 120 U/L), severe thrombocytopenia (platelet coun t = 50,000/µL) and isolated lymphopenia (500/µL), with normal renal function parameters (blood urea nitrogen = 20 mg/dL, creatinine = 0.83 mg/dL). The PCR COVID test turned out positive and confirmed the reinfection. Additionally, we assessed specific prognostic markers for COVID, and the results were as follows: C-reactive protein (1.91 mg/L, upper limit 3 mg/L), procalcitonin (0.59 ng/mL, upper limit 0.07 ng/mL), NT-proBNP (1621 pg/mL), Troponin I (19 ng/mL, cut-off value 29 ng/mL), ferritin (246 µg/L, upper limit 290 µg/L) and interleukin-6 (8.24 pg/mL, upper limit 7 pg/mL). The bacteriological screening at admission turned out negative.

**Figure 1:**
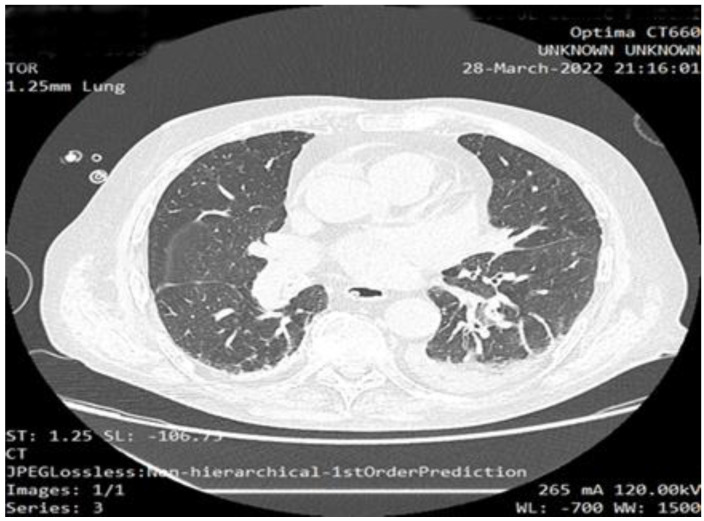
Native CT-scan revealing mild form of COVID-19 pneumonia.

The CT scan revealed mild COVID-19 pneumonia. The results are revealed in Figure 1.

The differential diagnosis included: pulmonary embolism (ruled out based on the chest CT scan), acute heart failure (no suggestive NT-proBNP increase, no peripheral edema, stable blood pressure, no structural changes specific to lung edema), bacterial pneumonia (bacteriological findings were negative, no specific structural changes), other causes for sepsis (negative bacteriological screening and absent clinical signs, with only isolated procalcitonin increase). The above-mentioned results pointed to a severe form of COVID-19 pneumonia in a patient with a relapsed myasthenic crisis.

The treatment strategies involved two main approaches: etiological and supportive treatment. The etiological treatment included: intravenous Remdesivir (Gilead Sciences Ireland UC, Carrigtwohill, Ireland) 200 mg/day and dexamethasone (E.I.P.I.CO MED SRL, Bucharest, Romania) 16 mg/day i.v. for 10 days. Additionally, five distinct sessions of plasma exchange (PLEX), each session with a plasma volume of approximately 4000 mL, were indicated.

Supportive therapy included: (1) non-invasive ventilation (NIV) alternating continuous positive airway pressure (CPAP) with biphasic positive airway pressure (BIPAP), adapted to blood gas analysis and patient comfort; (2) Neostigmine (ZENTIVA S.A., Bucharest, Romania) at an initial dose of 1 mg i.v. every 4 h, followed by 1 mg i.v. every 6 h, in accordance with the local protocol; (3) deep vein thrombosis prophylaxis with Enoxaparin (Sanofi Romania SRL, Bucharest, Romania) 40 mg/day subcutaneously; (4) Antiplatelet therapy with oral Clopidogrel (Sanofi-aventis Groupe, Paris, France) 75 mg/day for mixed primary prophylaxis of cerebral insult and myocardial infarction; (5) Stress ulcer prophylaxis with i.v. Omeprazole (Takeda GmbH, Konstanz, Germany) 40 mg/day, considering the mixed bleeding risks; (6) Beta-blocker therapy with oral Carvedilol (Hexal AG, Holzkirchen, Germany) 6.25 mg/day for tachycardia; (7) oral Escitalopram (H. Lundbeck A/S, Copenhagen, Denmark) 10 mg/day as antidepressant therapy, alongside with psychological support; and (8) nutritional support via nasojejunal tube.

The patient received 12 days of NIV, followed by three days of oxygen therapy via facial mask with a decremental flow ranging between 4–12 L/min. On the 15th day, the patient was discharged from the ICU. After another 20 days of integrated rehabilitation and specific neurological care, the patient was discharged from the hospital, with no long-term oxygen therapy.

## 3. Discussion

MC are most often triggered by infections; thus, COVID-19 pneumonia causes respiratory failure in MG patients via two separate mechanisms: firstly, through damage of the lung parenchyma, and secondly, via worsening of myasthenic symptoms [11,12,13]. Some of the drugs originally used in COVID-19 treatment (such as hydroxychloroquine or azithromycin) can also cause exacerbation of respiratory symptoms in myasthenia patients, either by way of increased acetylcholine receptor antibody production or by directly influencing neuromuscular transmission [14]. Although not the case for our patient, COVID-19-associated ARDS usually warrants the use of sedatives and neuromuscular blocking agents, which make the neurological evaluation of the patients difficult [12]. Moreover, the evolution of COVID-19 pneumonia in these patients is even more challenging to monitor due to the dampened inflammatory response in the context of long-term immunosuppressive therapy [15,16]. Outcomes in patients with concomitant COVID-19 pneumonia and MG are still a topic of debate. Kim et al. [17] reported that such patients have a higher risk of hospital and ICU admission than those without MG (even if suffering from other autoimmune conditions, such as rheumatoid arthritis or systemic lupus erythematosus), but mechanical ventilation requirements were not significantly different, and neither was the risk of death. A retrospective study by Digala et al. [18] noticed that the hospital length of stay was higher in myasthenic patients infected with SARS-CoV-2 than in those without. A systematic review by Abbas et al. [10] suggested that COVID-19 might worsen outcomes in MG cases, but it was not a definitive conclusion, as they lacked the required amount of data.

When managing MG patients with concomitant COVID-19 infection, one study has shown that long-term corticosteroids increase the risk of hospitalization with severe pneumonia, with the effect in direct relation to the dose, while recent rituximab administration increased the odds of death by a factor of over 35 [19].

In patients with MC, where a rapid improvement of muscle strength is required, one could resort to therapies such as IvIG or PLEX [20]. IvIG act by a variety of mechanisms, the most relevant in the context of MG being: (1) binding circulating antibodies; (2) inhibition of B-cell differentiation, migration, and antibody production; and (3) enhanced degradation of the complement system components, thus preventing the formation of membrane attack complexes [21]. PLEX works by separating the cellular and liquid components of blood, discarding the plasma, and replacing it with exogenous fluid (albumin solutions or healthy donor plasma); this way, the circulating antibodies are eliminated, improving the symptoms of MG. When performing a systematic literature review concerning variations in patient outcomes when treated with IvIG or PLEX, Ipe et al. [22] discovered that MC might be more responsive to PLEX than to intravenous immunoglobulin therapy, inducing a quicker, yet shorter-lived remission, leading to shorter ventilation times, with prolonged hospitalization. The literature seems to be remarkably sparse when tackling the topic of combined or consecutive IvIG and PLEX in patients with MC. Normally, an IvIG regimen dictates repeated doses at most every 3–4 weeks. Since our patient had recently received immunoglobulins for a different MC, he had to receive PLEX therapy, which is also proven to help in faster clinical recovery from COVID-19 pneumonia [23].

While the long-term outcomes of MG have been investigated in many studies, which do not cover the pathology addressed here (COVID and MG), we could conclude that the number of relapsing episodes represents an important risk factor for a detrimental long-term prognosis [24]. However, the prognosis of COVID-19 pneumonia in immunosuppressed patients appears to consist of prolonged viral shedding, even at levels lower than conventional assays’ detection limits, but not necessarily increased mortality [25,26,27,28].

## 4. Conclusions

COVID-19 pneumonia in patients with MC represents a real therapeutic challenge. The combined IvIG and PLEX at various time points could be beneficial for both pathologies, COVID-19 pneumonia, and myasthenia exacerbation, especially if the timeframe since the previous IvIG therapy is too short. Further clinical studies are needed in order to develop a rational therapeutic approach for the COVID-19 patients with autoimmune neuromuscular disorders.

## Data Availability

Not applicable.
